# Supplementary material for: Hot spot prediction in protein-protein interactions by an ensemble system
Source: BMC Syst Biol. 2018 Dec 31;12(Suppl 9):132. doi: 10.1186/s12918-018-0665-8 (PMC6311905; doi:10.1186/s12918-018-0665-8)
Supplement: Supplementary file 2 — All relevant MATLAB code. A simple Matlab implement of our predictor. (ZIP 19,742 kb) [file 12918_2018_665_MOESM2_ESM.zip › Additional file 2/Read ASA/ASA website/dbMPIKT/1U7FB.html]

 NetSurfP - Protein Surface Accessibility and Secondary Structure Predictions

  

|  |  |  |
| --- | --- | --- |
|  |  | NetSurfP - Protein Surface Accessibility and Secondary Structure Predictions  Technical University of Denmark |

  


---

```
# For publication of results, please cite:
# A generic method for assignment of reliability scores applied to solvent accessibility predictions.
# Bent Petersen, Thomas Nordahl Petersen, Pernille Andersen, Morten Nielsen and Claus Lundegaard
# BMC Structural Biology 2009, 9:51 doi:10.1186/1472-6807-9-51
#
# Column 1: Class assignment - B for buried or E for Exposed - Threshold: 25% exposure, but not based on RSA
# Column 2: Amino acid
# Column 3: Sequence name
# Column 4: Amino acid number
# Column 5: Relative Surface Accessibility - RSA
# Column 6: Absolute Surface Accessibility
# Column 7: Z-fit score for RSA prediction
# Column 8: Probability for Alpha-Helix
# Column 9: Probability for Beta-strand
# Column 10: Probability for Coil
E I  1U7FB                  1    0.755 139.638  -1.892   0.003   0.003   0.994
E S  1U7FB                  2    0.546  63.956  -1.548   0.018   0.047   0.935
E N  1U7FB                  3    0.699 102.260  -0.859   0.018   0.047   0.935
E H  1U7FB                  4    0.425  77.253  -0.492   0.018   0.047   0.935
E P  1U7FB                  5    0.648  91.994  -0.169   0.018   0.047   0.935
E A  1U7FB                  6    0.580  63.971   0.049   0.018   0.047   0.935
E P  1U7FB                  7    0.250  35.489   0.062   0.053   0.043   0.903
E E  1U7FB                  8    0.634 110.847   0.263   0.052   0.084   0.864
B Y  1U7FB                  9    0.224  47.933   0.229   0.064   0.216   0.721
B W  1U7FB                 10    0.093  22.342   0.304   0.074   0.484   0.442
B C  1U7FB                 11    0.024   3.398   0.606   0.021   0.756   0.223
B S  1U7FB                 12    0.204  23.874   0.749   0.072   0.855   0.073
B I  1U7FB                 13    0.027   5.032   1.059   0.072   0.855   0.073
B A  1U7FB                 14    0.055   6.061   0.957   0.072   0.855   0.073
B Y  1U7FB                 15    0.084  17.887   0.565   0.072   0.855   0.073
B F  1U7FB                 16    0.139  27.877   0.950   0.072   0.855   0.073
B E  1U7FB                 17    0.184  32.110   0.095   0.087   0.683   0.230
B M  1U7FB                 18    0.261  52.286   0.209   0.135   0.317   0.548
E D  1U7FB                 19    0.545  78.520  -0.758   0.052   0.084   0.864
E V  1U7FB                 20    0.285  43.743   0.440   0.056   0.142   0.802
E Q  1U7FB                 21    0.341  60.938   0.187   0.064   0.216   0.721
B V  1U7FB                 22    0.089  13.741   0.221   0.074   0.484   0.442
B G  1U7FB                 23    0.139  10.931   0.366   0.074   0.484   0.442
E E  1U7FB                 24    0.484  84.537  -0.011   0.074   0.484   0.442
B T  1U7FB                 25    0.234  32.456  -0.024   0.022   0.552   0.426
B F  1U7FB                 26    0.137  27.496   0.760   0.023   0.655   0.322
E K  1U7FB                 27    0.443  91.084   0.797   0.023   0.655   0.322
B V  1U7FB                 28    0.072  11.020  -1.058   0.022   0.552   0.426
E P  1U7FB                 29    0.352  49.963  -0.238   0.022   0.359   0.619
E S  1U7FB                 30    0.422  49.423  -1.012   0.019   0.141   0.840
E S  1U7FB                 31    0.648  75.957  -1.160   0.005   0.015   0.979
E C  1U7FB                 32    0.329  46.206  -0.398   0.005   0.015   0.979
B P  1U7FB                 33    0.193  27.401  -0.658   0.004   0.085   0.910
B I  1U7FB                 34    0.243  44.900   0.399   0.002   0.816   0.182
B V  1U7FB                 35    0.048   7.454   0.708   0.001   0.959   0.040
B T  1U7FB                 36    0.139  19.307   0.305   0.001   0.959   0.040
B V  1U7FB                 37    0.039   6.040  -0.759   0.001   0.900   0.099
B D  1U7FB                 38    0.164  23.690  -1.391   0.004   0.420   0.576
B G  1U7FB                 39    0.068   5.336  -1.439   0.021   0.279   0.699
B Y  1U7FB                 40    0.097  20.772  -1.516   0.004   0.514   0.481
B V  1U7FB                 41    0.225  34.582  -0.075   0.004   0.514   0.481
B D  1U7FB                 42    0.232  33.431  -1.133   0.004   0.197   0.799
B P  1U7FB                 43    0.286  40.555  -1.150   0.018   0.019   0.964
E S  1U7FB                 44    0.432  50.619  -2.360   0.016   0.005   0.979
E G  1U7FB                 45    0.607  47.739  -0.866   0.016   0.005   0.979
E G  1U7FB                 46    0.484  38.051  -2.817   0.003   0.003   0.994
E D  1U7FB                 47    0.387  55.767  -1.015   0.018   0.047   0.935
B R  1U7FB                 48    0.118  27.045  -1.336   0.004   0.420   0.576
B F  1U7FB                 49    0.044   8.811  -0.323   0.023   0.655   0.322
B C  1U7FB                 50    0.034   4.830  -1.702   0.023   0.655   0.322
B L  1U7FB                 51    0.032   5.951  -0.668   0.021   0.451   0.528
B G  1U7FB                 52    0.065   5.115  -1.375   0.021   0.279   0.699
B Q  1U7FB                 53    0.126  22.504  -1.868   0.020   0.205   0.775
B L  1U7FB                 54    0.108  19.738  -1.037   0.019   0.141   0.840
E S  1U7FB                 55    0.320  37.445  -0.860   0.018   0.088   0.893
B N  1U7FB                 56    0.222  32.515  -0.753   0.018   0.047   0.935
B V  1U7FB                 57    0.248  38.041  -1.105   0.018   0.019   0.964
E H  1U7FB                 58    0.402  73.069  -1.184   0.005   0.015   0.979
B R  1U7FB                 59    0.173  39.663  -0.097   0.058   0.017   0.925
E T  1U7FB                 60    0.465  64.565  -0.439   0.246   0.004   0.750
E E  1U7FB                 61    0.561  98.059  -0.305   0.782   0.003   0.216
E A  1U7FB                 62    0.291  32.046   0.334   0.858   0.002   0.139
B I  1U7FB                 63    0.046   8.436  -0.045   0.923   0.002   0.076
E E  1U7FB                 64    0.471  82.266   1.311   0.938   0.007   0.055
E R  1U7FB                 65    0.488 111.683   1.492   0.938   0.007   0.055
B A  1U7FB                 66    0.036   4.011  -0.201   0.879   0.010   0.111
B R  1U7FB                 67    0.216  49.510   0.539   0.879   0.010   0.111
E L  1U7FB                 68    0.528  96.750   0.803   0.831   0.044   0.125
E H  1U7FB                 69    0.312  56.716   1.557   0.751   0.050   0.199
B I  1U7FB                 70    0.071  13.154   0.329   0.502   0.102   0.396
E G  1U7FB                 71    0.313  24.641  -0.681   0.113   0.087   0.800
E K  1U7FB                 72    0.435  89.562   0.860   0.018   0.088   0.893
B G  1U7FB                 73    0.185  14.575  -1.906   0.021   0.451   0.528
B V  1U7FB                 74    0.039   6.040   0.610   0.002   0.816   0.182
B Q  1U7FB                 75    0.255  45.525   1.353   0.001   0.959   0.040
B L  1U7FB                 76    0.049   8.899   0.831   0.001   0.959   0.040
B E  1U7FB                 77    0.222  38.783   0.475   0.001   0.959   0.040
B C  1U7FB                 78    0.194  27.308   0.895   0.001   0.900   0.099
E K  1U7FB                 79    0.368  75.780   0.938   0.003   0.718   0.279
E G  1U7FB                 80    0.693  54.555  -1.134   0.004   0.197   0.799
E E  1U7FB                 81    0.646 112.786   0.176   0.005   0.045   0.951
B G  1U7FB                 82    0.159  12.482  -0.919   0.004   0.138   0.858
E D  1U7FB                 83    0.309  44.512   0.963   0.004   0.616   0.381
B V  1U7FB                 84    0.028   4.350   0.685   0.001   0.959   0.040
B W  1U7FB                 85    0.095  22.751   1.060   0.006   0.962   0.032
B V  1U7FB                 86    0.020   3.043   0.944   0.006   0.962   0.032
B R  1U7FB                 87    0.174  39.892   0.843   0.001   0.959   0.040
B C  1U7FB                 88    0.039   5.504  -0.226   0.002   0.816   0.182
B L  1U7FB                 89    0.165  30.248   0.370   0.005   0.262   0.733
B S  1U7FB                 90    0.085   9.927  -1.855   0.005   0.045   0.951
E D  1U7FB                 91    0.627  90.322   0.262   0.005   0.045   0.951
B H  1U7FB                 92    0.103  18.827  -0.169   0.005   0.045   0.951
B A  1U7FB                 93    0.152  16.772  -0.025   0.005   0.262   0.733
B V  1U7FB                 94    0.034   5.195  -0.258   0.002   0.816   0.182
B F  1U7FB                 95    0.054  10.838  -0.109   0.001   0.900   0.099
B V  1U7FB                 96    0.031   4.765   0.145   0.011   0.918   0.071
B Q  1U7FB                 97    0.210  37.488  -0.319   0.022   0.552   0.426
B S  1U7FB                 98    0.069   8.122  -0.173   0.019   0.141   0.840
B Y  1U7FB                 99    0.278  59.515  -0.346   0.019   0.141   0.840
E Y  1U7FB                100    0.327  69.859  -1.259   0.019   0.141   0.840
B L  1U7FB                101    0.109  19.958  -0.330   0.052   0.084   0.864
E D  1U7FB                102    0.269  38.835  -0.961   0.053   0.043   0.903
E R  1U7FB                103    0.423  96.844  -0.192   0.113   0.043   0.844
E E  1U7FB                104    0.537  93.779  -0.976   0.113   0.043   0.844
E A  1U7FB                105    0.498  54.847  -2.275   0.058   0.017   0.925
E G  1U7FB                106    0.454  35.690  -2.095   0.018   0.019   0.964
E R  1U7FB                107    0.401  91.760  -1.221   0.053   0.043   0.903
E A  1U7FB                108    0.357  39.352  -0.689   0.053   0.043   0.903
E P  1U7FB                109    0.413  58.548  -1.031   0.053   0.043   0.903
E G  1U7FB                110    0.504  39.696  -1.116   0.018   0.047   0.935
E D  1U7FB                111    0.343  49.354  -0.707   0.056   0.142   0.802
B A  1U7FB                112    0.246  27.076   0.294   0.021   0.451   0.528
B V  1U7FB                113    0.174  26.790  -0.278   0.021   0.756   0.223
B H  1U7FB                114    0.149  27.194   0.862   0.021   0.756   0.223
E K  1U7FB                115    0.312  64.240   0.120   0.021   0.756   0.223
B I  1U7FB                116    0.064  11.803   0.385   0.021   0.451   0.528
B Y  1U7FB                117    0.226  48.189   0.732   0.005   0.045   0.951
E P  1U7FB                118    0.421  59.811  -1.851   0.005   0.015   0.979
E S  1U7FB                119    0.484  56.678  -1.986   0.018   0.019   0.964
B A  1U7FB                120    0.234  25.798  -0.772   0.019   0.141   0.840
B Y  1U7FB                121    0.224  47.783  -0.260   0.021   0.451   0.528
B I  1U7FB                122    0.075  13.949   0.710   0.023   0.655   0.322
B K  1U7FB                123    0.242  49.821   0.178   0.021   0.756   0.223
B V  1U7FB                124    0.053   8.115  -0.027   0.023   0.655   0.322
B F  1U7FB                125    0.122  24.445   0.262   0.021   0.451   0.528
E D  1U7FB                126    0.400  57.611   0.683   0.056   0.142   0.802
B L  1U7FB                127    0.198  36.290   0.314   0.522   0.016   0.462
E R  1U7FB                128    0.590 135.018   1.129   0.879   0.010   0.111
E Q  1U7FB                129    0.428  76.352   1.264   0.923   0.002   0.076
B C  1U7FB                130    0.067   9.407  -0.539   0.923   0.002   0.076
B H  1U7FB                131    0.268  48.695   0.447   0.970   0.001   0.030
E R  1U7FB                132    0.558 127.828   1.282   0.970   0.001   0.030
B Q  1U7FB                133    0.239  42.703   0.213   0.923   0.002   0.076
B M  1U7FB                134    0.123  24.692  -0.276   0.923   0.002   0.076
E Q  1U7FB                135    0.443  79.048   0.172   0.858   0.002   0.139
E Q  1U7FB                136    0.528  94.247  -0.166   0.782   0.003   0.216
E Q  1U7FB                137    0.371  66.314  -0.588   0.694   0.003   0.303
B A  1U7FB                138    0.294  32.344  -1.494   0.694   0.003   0.303
E A  1U7FB                139    0.492  54.218  -1.216   0.502   0.002   0.495
E T  1U7FB                140    0.535  74.135  -0.263   0.430   0.016   0.555
E A  1U7FB                141    0.361  39.793  -0.969   0.339   0.016   0.645
E Q  1U7FB                142    0.342  61.135   0.003   0.430   0.016   0.555
E A  1U7FB                143    0.433  47.684  -0.818   0.802   0.014   0.185
B A  1U7FB                144    0.298  32.818  -1.137   0.879   0.010   0.111
E A  1U7FB                145    0.373  41.072  -0.988   0.879   0.010   0.111
B A  1U7FB                146    0.280  30.878  -0.752   0.802   0.014   0.185
E A  1U7FB                147    0.369  40.675  -0.913   0.802   0.014   0.185
E Q  1U7FB                148    0.432  77.119  -0.353   0.717   0.014   0.269
E A  1U7FB                149    0.406  44.686  -0.450   0.622   0.015   0.363
E A  1U7FB                150    0.326  35.914  -1.371   0.339   0.016   0.645
B A  1U7FB                151    0.310  34.173  -0.891   0.354   0.048   0.598
E V  1U7FB                152    0.341  52.412  -0.743   0.354   0.048   0.598
E A  1U7FB                153    0.481  52.984  -1.371   0.278   0.093   0.628
E G  1U7FB                154    0.504  39.673  -1.960   0.184   0.043   0.773
E N  1U7FB                155    0.433  63.347  -1.071   0.184   0.043   0.773
E I  1U7FB                156    0.308  56.924  -1.605   0.113   0.043   0.844
E P  1U7FB                157    0.499  70.865  -1.835   0.113   0.043   0.844
E G  1U7FB                158    0.318  25.050  -2.504   0.058   0.017   0.925
B P  1U7FB                159    0.304  43.109  -2.728   0.053   0.043   0.903
E G  1U7FB                160    0.469  36.934  -2.497   0.115   0.016   0.868
B S  1U7FB                161    0.272  31.878  -1.457   0.115   0.016   0.868
E V  1U7FB                162    0.382  58.667  -2.414   0.181   0.016   0.803
E G  1U7FB                163    0.481  37.878  -2.008   0.257   0.016   0.727
E G  1U7FB                164    0.365  28.725  -1.602   0.339   0.016   0.645
B I  1U7FB                165    0.292  54.057  -1.322   0.430   0.016   0.555
E A  1U7FB                166    0.316  34.867  -1.256   0.455   0.046   0.498
E P  1U7FB                167    0.396  56.207  -1.041   0.455   0.046   0.498
E A  1U7FB                168    0.406  44.719  -1.643   0.455   0.046   0.498
E I  1U7FB                169    0.414  76.553  -1.227   0.455   0.046   0.498
E S  1U7FB                170    0.432  50.630  -1.211   0.455   0.046   0.498
B L  1U7FB                171    0.283  51.891  -0.935   0.354   0.048   0.598
B S  1U7FB                172    0.198  23.159  -0.373   0.354   0.048   0.598
E A  1U7FB                173    0.480  52.874  -0.244   0.455   0.046   0.498
E A  1U7FB                174    0.417  45.997  -1.424   0.455   0.046   0.498
B A  1U7FB                175    0.218  23.968  -1.207   0.354   0.048   0.598
E G  1U7FB                176    0.409  32.157  -1.915   0.113   0.087   0.800
B I  1U7FB                177    0.268  49.580  -0.316   0.113   0.087   0.800
E G  1U7FB                178    0.335  26.364  -1.107   0.191   0.086   0.723
B V  1U7FB                179    0.191  29.295  -0.370   0.561   0.047   0.393
E D  1U7FB                180    0.386  55.637  -0.854   0.660   0.049   0.291
E D  1U7FB                181    0.319  45.925  -0.057   0.622   0.015   0.363
B L  1U7FB                182    0.058  10.528  -0.150   0.717   0.014   0.269
E R  1U7FB                183    0.288  66.044   0.383   0.751   0.050   0.199
E R  1U7FB                184    0.372  85.119   0.385   0.660   0.049   0.291
B L  1U7FB                185    0.146  26.769   0.922   0.605   0.105   0.290
B C  1U7FB                186    0.023   3.243   0.473   0.352   0.332   0.316
B I  1U7FB                187    0.102  18.962   0.291   0.169   0.612   0.219
B L  1U7FB                188    0.037   6.848  -0.297   0.157   0.773   0.069
B R  1U7FB                189    0.075  17.244   0.008   0.157   0.773   0.069
B M  1U7FB                190    0.037   7.304  -0.177   0.157   0.773   0.069
B S  1U7FB                191    0.075   8.790  -0.438   0.265   0.667   0.069
B F  1U7FB                192    0.051  10.216  -0.040   0.273   0.587   0.140
B V  1U7FB                193    0.037   5.702  -0.599   0.252   0.423   0.325
E K  1U7FB                194    0.363  74.669   0.279   0.216   0.235   0.548
E G  1U7FB                195    0.366  28.788  -1.860   0.052   0.084   0.864
B W  1U7FB                196    0.259  62.386  -0.452   0.053   0.043   0.903
E G  1U7FB                197    0.361  28.395  -0.380   0.018   0.047   0.935
E P  1U7FB                198    0.610  86.516  -0.838   0.053   0.043   0.903
E D  1U7FB                199    0.589  84.846  -0.349   0.113   0.043   0.844
B Y  1U7FB                200    0.122  26.157   0.866   0.053   0.043   0.903
E P  1U7FB                201    0.572  81.124   0.484   0.058   0.017   0.925
E R  1U7FB                202    0.337  77.265   0.268   0.053   0.043   0.903
E Q  1U7FB                203    0.503  89.872  -0.317   0.053   0.043   0.903
E S  1U7FB                204    0.380  44.524   0.603   0.052   0.084   0.864
B I  1U7FB                205    0.085  15.651  -0.831   0.056   0.142   0.802
E K  1U7FB                206    0.309  63.623  -0.081   0.056   0.142   0.802
E E  1U7FB                207    0.505  88.171   0.021   0.056   0.142   0.802
B T  1U7FB                208    0.072   9.986   0.345   0.053   0.043   0.903
B P  1U7FB                209    0.079  11.168   0.176   0.052   0.084   0.864
B C  1U7FB                210    0.029   4.044   0.280   0.159   0.516   0.325
B W  1U7FB                211    0.049  11.736   0.552   0.157   0.773   0.069
B I  1U7FB                212    0.028   5.180   0.316   0.151   0.816   0.033
B E  1U7FB                213    0.066  11.583   0.501   0.151   0.816   0.033
B I  1U7FB                214    0.022   4.070   1.099   0.151   0.816   0.033
B H  1U7FB                215    0.132  23.993   1.036   0.157   0.773   0.069
B L  1U7FB                216    0.053   9.723  -0.026   0.373   0.401   0.226
B H  1U7FB                217    0.184  33.524   0.157   0.561   0.047   0.393
B R  1U7FB                218    0.164  37.625   0.016   0.522   0.016   0.462
B A  1U7FB                219    0.051   5.664  -0.199   0.802   0.014   0.185
B L  1U7FB                220    0.086  15.783   0.237   0.938   0.007   0.055
E Q  1U7FB                221    0.352  62.831   0.373   0.923   0.002   0.076
B L  1U7FB                222    0.108  19.702  -0.131   0.970   0.001   0.030
B L  1U7FB                223    0.036   6.628   0.081   0.970   0.001   0.030
B D  1U7FB                224    0.206  29.713  -0.270   0.970   0.001   0.030
E E  1U7FB                225    0.391  68.273   1.015   0.970   0.001   0.030
B V  1U7FB                226    0.065   9.960  -0.031   0.970   0.001   0.030
B L  1U7FB                227    0.130  23.876   0.305   0.923   0.002   0.076
E H  1U7FB                228    0.601 109.358   0.260   0.782   0.003   0.216
E T  1U7FB                229    0.435  60.307   0.422   0.600   0.003   0.397
B M  1U7FB                230    0.135  27.054   0.476   0.109   0.005   0.886
E P  1U7FB                231    0.458  65.004  -1.891   0.016   0.005   0.979
E I  1U7FB                232    0.311  57.479  -1.203   0.003   0.003   0.994
E A  1U7FB                233    0.583  64.214  -2.442   0.003   0.003   0.994
E D  1U7FB                234    0.546  78.722  -0.877   0.003   0.003   0.994
E P  1U7FB                235    0.519  73.575  -0.926   0.003   0.003   0.994
E Q  1U7FB                236    0.515  92.068  -0.693   0.003   0.003   0.994
E P  1U7FB                237    0.439  62.322  -1.425   0.005   0.015   0.979
E L  1U7FB                238    0.392  71.848  -1.058   0.005   0.015   0.979
E D  1U7FB                239    0.923 133.062  -0.208   0.000   0.001   0.999

Explain the output.  Go back.
```

---
